# Physician vaccination practices in mild to moderate inborn errors of immunity and retrospective review of vaccine completeness in IEI: results from the Canadian Immunization Research Network

**DOI:** 10.1186/s13223-022-00667-1

**Published:** 2022-04-09

**Authors:** Sneha Suresh, Joseline Zafack, Anne Pham-Huy, Beata Derfalvi, Manish Sadarangani, Athena McConnell, Bruce Tapiéro, Scott A. Halperin, Gaston De Serres, Jeffrey M Pernica, Karina A. Top

**Affiliations:** 1 Division of Immunology, Department of Pediatrics, Edmonton Clinic Health Academy, 3-529, 11405 87 Ave, Edmonton, AB T6G 1C9 Canada; 2 Division of Infectious Disease and IHOPE, Department of Paediatrics, Stollery Children’s Hospital, University of Alberta, Edmonton, Canada; 3 Public Health Agency of Canada, Ottawa, Canada; 4 Division of Infectious Diseases, Immunology and Allergy, Department of Paediatrics, Children’s Hospital of Eastern Ontario, University of Ottawa, Ottawa, Canada; 5 Division of Immunology, Departments of Paediatrics and Microbiology and Immunology, IWK Health Centre, Dalhousie University, Halifax, Canada; 6 Vaccine Evaluation Center, BC Children’s Hospital Research Institute, Department of Pediatrics, University of British Columbia, Vancouver, Canada; 7 Division of Infectious Diseases, Department of Pediatrics, Jim Pattison Children’s Hospital, University of Saskatchewan, Saskatoon, Canada; 8 Division of Infectious Diseases, Department of Pediatrics, CHU Sainte Justine, Université de Montreal, Montreal, Canada; 9 Departments of Paediatrics and Microbiology and Immunology, Canadian Center for Vaccinology IWK Health Centre, Dalhousie University, Halifax, Canada; 10 Department of Social and Preventive Medicine, Institut Nationale de Santé Publique du Québec, Université Laval, Québec, Canada; 11 Division of Infectious Diseases, Department of Pediatrics, McMaster University, Hamilton, Canada; 12 Departments of Pediatrics and Community Health and Epidemiology, Canadian Center for Vaccinology, IWK Health Centre, Dalhousie University, Halifax, Canada

**Keywords:** B cell deficiency, Combined immunodeficiency, Immunization, Primary antibody deficiency, Vaccination, Vaccine coverage

## Abstract

**Background and objectives:**

Safety and effectiveness concerns may preclude physicians from recommending vaccination in mild/moderate inborn errors of immunity (IEI). This study describes attitudes and practices regarding vaccination among physicians who care for patients with mild/moderate B cell or mild/moderate combined immunodeficiencies (CID) and vaccination completeness among patients diagnosed with IEIs.

**Methods:**

Canadian physicians caring for children with IEI were surveyed about attitudes and practices regarding vaccination in mild/moderate IEI. Following informed consent, immunization records of pediatric patients with IEI evaluated before 7 years of age were reviewed. Vaccine completeness was defined at age 2 years as 4 doses of diphtheria-tetanus-pertussis (DTaP), 3 doses pneumococcal conjugate (PCV), and 1 dose measles-mumps-rubella (MMR) vaccines. At 7 years 5 doses of DTP and 2 doses MMR were required.

**Results:**

Forty-five physicians from 8 provinces completed the survey. Most recommended inactivated vaccines for B cell deficiency: (84% (38/45) and CID (73% (33/45). Fewer recommended live attenuated vaccines (B cell: 53% (24/45), CID 31% (14/45)). Of 96 patients with IEI recruited across 7 centers, vaccination completeness at age 2 was 25/43 (58%) for predominantly antibody, 3/13 (23%) for CID, 7/35 (20%) for CID with syndromic features, and 4/4 (100%) for innate/phagocyte defects. Completeness at age 7 was 15%, 17%, 5%, and 33%, respectively.

**Conclusion:**

Most physicians surveyed recommended inactivated vaccines in children with mild to moderate IEI. Vaccine completeness for all IEI was low, particularly at age 7. Further studies should address the reasons for low vaccine uptake among children with IEI and whether those with mild-moderate IEI, where vaccination is recommended, eventually receive all indicated vaccines.

**Supplementary Information:**

The online version contains supplementary material available at 10.1186/s13223-022-00667-1.

## Background

A significant cornerstone of the management of inborn errors of immunity (IEIs) is prevention of infection, and immunization is an important method of protection. This typically is achieved through passive immunization (with hyperimmunoglobulin or polyclonal immunoglobulin) or vaccination.

For profound IEIs such as agammaglobulinemia or severe combined immunodeficiency (SCID), passive immunization with regular polyclonal immunoglobulin is indicated as standard of care [1]. For mild-moderate B cell and combined immunodeficiencies (CIDs), vaccination can be considered. Currently, only DiGeorge syndrome and Cartilage hair-hypoplasia have systematically accrued vaccine safety data to guide clinicians [2–6]. In other mild-moderate IEI where a certain degree of antibody function and/or cell-mediated immunity is preserved and for which immunoglobulin replacement therapy is not clinically indicated, the decision of which vaccine(s) to administer can be difficult, given concerns regarding safety, immunogenicity and effectiveness [1].

Safety is a main consideration prior to vaccination in patients with IEI [1]. Adverse events after live attenuated vaccination have been documented in patients with a range of IEIs [7–22]. National guidelines, based on cohort studies, case series and expert opinion offer some advice; however, high-quality evidence regarding best practice is lacking [1, 23–27]. The clinical heterogeneity of certain IEIs can make generalized recommendations difficult to both create and apply. Finally, patients with suspected IEI are sometimes told to avoid live vaccinations until diagnostic work up is complete. This may lead to vaccine hesitancy even after a profound IEI is ruled out and live vaccines are indicated.

These evidence gaps create potential for practice variation and low vaccination rates. The objectives of this study were to describe attitudes and practices regarding immunization among Immunologists, Pediatricians and Infectious disease specialists who care for patients with mild/moderate B cell defects and CIDs, and to estimate vaccine completeness among patients with IEI at 2 and 7 years of age.

## Methods

### Physician survey

A self-administered online questionnaire was developed on SurveyMonkey® and distributed to Pediatricians, Pediatric infectious disease physicians and Immunologists at tertiary care pediatric centers across Canada via the Canadian Society of Allergy and Clinical Immunology (CSACI) physician membership list, as well as the Special Immunization Network (SIC), a research network focused on immunization practices for immunocompromised patients [28]. The sample of physicians was identified by the co-authors (APH, MS, AM, BT, BD, SAH, GDS, JP, KAT) by using local hospital listings or medical association records to find physicians who manage patients with IEIs at Canadian pediatric tertiary care centres. With consent, the survey link was distributed via email from May to September 2017, with reminders sent 15 and 30 days after the initial email.

The questionnaire collected information on demographics, practice setting, specialty, number of IEI patients followed and perceptions of safety and effectiveness of specific vaccines, practices and recommendations regarding specific live and inactivated vaccines for patients with mild/moderate B or combined immunodeficiencies, as well as factors that influenced their vaccination recommendations and investigations conducted (e.g., B and T cell enumeration, vaccine-specific antibody titers). Factors surveyed were decided via expert consensus. Survey questions are included in supplementary material.

The following categories of immunodeficiency were used:

- “mild/moderate primary B cell defects”—mild/moderate primary humoral defects (e.g. IgA deficiency, IgG subclass deficiency, specific antibody deficiency, transient hypogammaglobulinemia of infancy) and other unspecified or syndrome-related mild/moderate primary hypogammaglobulinemia (e.g. Down syndrome).
- “partial (mild/moderate) primary combined immunodeficiencies (CID)”—CIDs characterized by an incomplete reduction in T-cell number or activity; where part of the immune system’s ability to respond to infectious organisms is retained (e.g. Di George syndrome, Ataxia-telangiectasia, Wiskott-Aldrich syndrome, early purine nucleoside phosphorylase deficiency).

### Retrospective review of vaccination completeness in pediatric IEI patients

All 11 SIC network sites were approached to participate in the retrospective chart review, and seven sites with active IEI clinics participated. Research ethics approval was obtained at each site. All patients aged < 18 years followed for IEI between January 1st, 2004 and December 31st, 2016 were identified from hospital records, appointment listings and clinic databases.

Inclusion criteria were: diagnostic workup of IEI before 7th birthday and one of the following types of IEI, according to the IUIS classification at the time of survey [29, 30]:

- Immunodeficiencies affecting cellular and humoral immunity
- Combined immunodeficiency with associated or syndromic features
- Predominantly antibody deficiencies
- Defects of intrinsic and innate immunity and congenital defects of phagocyte number or function

Exclusion criteria were:

- History of hematopoietic stem cell transplant
- Not alive at time of survey (due to the sensitivity of contacting parents for consent and vaccination records)
- Caregiver did not provide informed consent

The study consent form and a questionnaire to capture immunization history were mailed to caregivers of eligible patients. Research staff obtained verbal consent and collected vaccination information from caregiver records. All vaccination histories were crosschecked against public health records and vaccine provider records. Medical record abstraction was performed by a research nurse using standardized forms to capture details regarding IEI diagnosis, laboratory investigations, and treatment.

### Data analysis

For the physician survey, analysis was descriptive with responses reported as counts and proportions. Five-point Likert scales were collapsed into the following categories for analysis:

- very/somewhat effective or safe
- very/somewhat ineffective or unsafe
- don’t know
- always/often/sometimes recommend
- rarely/never recommend

Respondents who did not answer the question were excluded from analysis to differentiate them from respondents who answered “don’t know”.

For the retrospective review, the primary outcome was vaccination completeness. Vaccination completeness was defined to encompass the variability in the vaccination schedules in various Canadian provinces (see Table 1). Overall completeness was defined as receipt of all recommended inactivated and live vaccines, not including influenza vaccine. Vaccine completeness was reported for the 5 vaccine types by category of IEI. Proportions were compared using Chi square or Fisher exact tests when indicated. Statistical testing was bilateral with significance set at p < 0.05. No adjustment was made for multiple comparisons. Spearman correlation was undertaken to study correlation between investigations undertaken by physicians and attitudes towards vaccination.

**Table 1:** Minimum requirements for vaccine completeness*

Vaccine	Abbreviation	Minimum requirement
Diphtheria, Tetanus, acellular Pertussis, Haemophilus influenzae B, inactivated polio vaccine ± hepatitis B vaccine	DTaP-Hib-IPV or DTaP-HBV-Hib-IPV	• 4 doses by 2 years of age • 1 dose of dTap-IPV or DTaP-IPV administered on/after 4 years of age and by 7 years of age
Pneumococcal conjugate vaccine	PCV7, 10, or 13	• 1 dose given on/after 12 months of age • and/or 3 doses by 2 years of age
Measles, mumps, rubella ± varicella vaccine:	MMRV or MMR + V	• 1 dose by 2 years of age • And 2 doses by 7 years of age
Meningococcal conjugate vaccine or meningococcal conjugate C	Men-C-ACWY or Men-C–C	• 1 dose at any age
Influenza	IIV	• 1 dose in the past year • And ≥ 3 doses in last 5 years

^*^Overall completeness was defined based on DTaP-Hib-IPV, PCV, MMRV, Men-C vaccination status

## Results

### Physicians’ attitudes and practices

Of 50 physicians who were invited to participate in the survey, 46 (92%) responded (Table 2). One Infectious disease specialist (IDS) who did not follow patients with IEI was excluded. Among the remaining 45, there were 23 Immunologists, 19 IDS, and 3 general Pediatricians. Physicians were located in 8 of 10 Canadian provinces with the majority of respondents based in Quebec (39%, 18/45) and British Columbia (24%, 11/45). All respondents practiced in university-affiliated settings and most were hospital-based (90%, 38/45). Overall, 73% (33/45) of physicians were in practice for ≥ 5 years. Immunologists were more likely than IDS to have seen more than 10 B cell or CID patients in the past 12 months (B cell: 11%, 2/19 versus 57% 13/23, p = 0.006; CID: 11% (2/19) versus 48% (11/23), p = 0.014).

**Table 2:** Description of physician respondents’ profiles and practice settings

	Pediatrician N = 3	IDS N = 19	Immunologist N = 23
*On what qualification do you follow children with B or T cell deficiency?*			
Consultant^a^	0	14 (78%)	8 (35%)
Attending physician^b^	1 (33%)	1 (5%)	1 (4%)
Both consultant and attending physician	2 (67%)	3 (17%)	14 (61%)
Did not answer	0	1	0
*Where is your main practice located (Province/Territory)?*			
Alberta	0	0	1 (4%)
British Columbia	0	5 (26%)	6 (26%)
Manitoba	0	0	1 (4%)
Nova Scotia	0	0	2 (9%)
Newfoundland	0	0	3 (13%)
Ontario	0	2 (11%)	2 (9%)
Quebec	0	10 (53%)	8 (35%)
Saskatchewan	3 (100%)	2 (11%)	0
*How many years have you been in practice?*			
0 to 4 years	0	2 (11%)	6 (27%)
5 to 9 years	2 (67%)	4 (22%)	4 (18%)
10 to 19 years	1 (33%)	5 (28%)	7 (32%)
20 to 29 years	0	4 (22%)	1 (5%)
≥ 30 years	0	3 (17%)	4 (18%)
Did not answer	0	1	1
*Which of the following best describes your practice setting?*			
Hospital-based	3 (100%)	19 (100%)	19 (83%)
Community-based	0	0	3 (13%)
Both academic and community hospital	0	0	1 (4%)
*Is your practice setting university affiliated?*			
Yes	3 (100%)	19 (100%)	23 (100%)
No	0	0	0
*In the past 12 months, how many patients with mild/moderate B cell deficiency did you see?*			
0	2 (67%)	2 (10%)	0
1–5	1 (33%)	12 (60%)	6 (26%)
6–10	0	3 (15%)	4 (17%)
≥ 11	0	2 (10%)	13 (57%)
*In the past 12 months, how many patients with mild/moderate T cell deficiency did you see?*			
0	1 (33%)	2 (10%)	1 (4%)
1–5	1 (33%)	12 (60%)	5 (22%)
6–10	1 (33%)	3 (15%)	6 (26%)
≥ 11	0 (33%)	2 (10%)	11 (48%)
*What information sources do you use to answer immunization-related questions? *^*c*^			
Provincial Immunization Guide	0	16 (84%)	16 (84%)
Canadian Immunization Guide	2 (67%)	13 (68%)	15 (65%)
Colleagues	2 (67%)	13 (68%)	12 (52%)
Red book	3 (100%)	12 (63%)	13 (68%)
CDC website	0	8 (42%)	11 (48%)
Other sources (number of physicians who mentioned the source) ^c, d^	0	5 (26%)	4 (17%)
*Total number of sources cited*			
≤ 2 sources	2 (67%)	5 (26%)	9 (39%)
3–4 sources	1 (33%)	6 (32%)	8 (35%)
≥ 5 sources	0	8 (42%)	6 (26%)
*In general, are vaccines administered to patients with mild/moderate B or T cell defects at your practice setting?*			
Yes	3 (100%)	15 (79%)	21 (91%)
No	0	3 (16%)	2 (9%)
Did not answer	0	1 (5%)	0

“Mild/moderate primary B cell defects”: Clearly defined mild to moderate primary humoral defects (e.g. IgA deficiency, IgG subclass deficiency, specific antibody deficiency, transient hypogammaglobulinemia of infancy) and other unspecified or syndrome-related mild/moderate primary hypogammaglobulinemia (e.g. Down syndrome)

“Mild/moderate combined defects (CID)”: Characterized by an incomplete reduction in T-cell number or activity where part of the immune system’s ability to respond to infectious organisms is retained (e.g. partial DiGeorge syndrome, Ataxia Telangiectasia, Wiskott Aldrich syndrome, Purine Nucleoside Phosphorylase Deficiency)

*AAAAI* American Academy of Allergy Asthma and Immunology, *CDC* Center for Disease Control and Prevention, *CIS* clinical immunology society, *HIV* human immunodeficiency virus, *ID* infectious disease, *IDSA* infectious diseases society of America, *JACI* journal of allergy and clinical immunology

^a^Consultant: physician that gives a one-time opinion on the patient’s management

^b^Attending physician: physician in charge of regular follow up

^c^Respondents could give more than one response

^d^Other sources: IDSA Guidelines, AAAAI Practice Parameters, Medline or articles, Journal of allergy and clinical immunology, primary literature, CIS Listservs and Immunology Conferences

Physicians’ perceptions of the effectiveness and safety of 5 specific vaccines in patients with mild/moderate B cell deficiencies and CID are shown in Table 3. There were no significant differences in perceived effectiveness of inactivated influenza vaccine and live attenuated influenza vaccine (LAIV) (Table 3). Most respondents considered inactivated vaccines to be safe for patients with B cell and CID, but less than half indicated live vaccines were “very or somewhat safe”, for patients with mild/moderate CID.

**Table 3:** Perceptions of vaccine effectiveness and safety for B cell deficiency and CID

Vaccine effectiveness (N = 45)						Vaccine safety (N = 45)				
	Very/somewhat effective	Very/somewhat ineffective	Don’t know	Respondents that answered question (n)	Respondents that did not answer* (n)	Very/somewhat safe	Very/somewhat unsafe	Don’t know	Respondents that answered question (n)	Respondents that did not answer* (n)
*B cell IEI*										
DTaP/Tdap	42% (19)	7% (3)	29% (13)	35	10	69% (31)	0% (0)	7% (3)	34	11
Influenza (IIV)	36% (16)	7% (3)	33% (15)	34	11	62% (28)	4% (2)	7% (3)	33	12
Influenza (LAIV)	22% (10)	9% (4)	44% (20)	34	11	36% (16)	22% (10)	18% (8)	34	11
MMR/Varicella/MMRV	31% (14)	11% (5)	36% (16)	35	10	38% (17)	24% (11)	13% (6)	34	11
Rotavirus	22% (10)	11% (5)	44% (20)	35	10	36% (16)	16% (7)	18% (8)	31	14
*CID IEI*										
DTaP/Tdap	44% (20)	9% (4)	22% (10)	34	11	67% (30)	2% (1)	4% (2)	33	12
Influenza (IIV)	40% (18)	9% (4)	24% (11)	33	12	58% (26)	4% (2)	11% (5)	33	12
Influenza (LAIV)	13% (6)	16% (7)	44% (20)	33	12	13% (6)	40% (18)	20% (9)	35	10
MMR/Varicella/MMRV	24% (11)	18% (8)	29% (13)	32	13	16% (7)	49% (22)	9% (4)	33	12
Rotavirus	16% (7)	11% (5)	44% (20)	32	13	13% (6)	38% (17)	20% (9)	32	13

*Imm* Immunologists, *IDS* Infectious Disease Specialists, *MMR* Measles/Mumps/Rubella Vaccine, *MMRV* Measles/Mumps/Rubella/Varicella Vaccine, *DTaP-Hib-IPV* Diptheria/Tetanus/acellular pertussis/Haemophilus Influenzae B/Inactivated Polio Vaccine, *DTaP* Diphtheria/Tetanus/Acellular Pertussis Vaccine, *TdaP* Tetanus/lower dose Diphtheria/Acellular Pertussis Vaccine, *IIV* Inactivated Influenza Vaccine, *LAIV* Live Attenuated Influenza Vaccine

“Mild/moderate primary B cell defects”: Clearly defined mild to moderate primary humoral defects (e.g. IgA deficiency, IgG subclass deficiency, specific antibody deficiency, transient hypogammaglobulinemia of infancy) and other unspecified or syndrome-related mild/moderate primary hypogammaglobulinemia (e.g. Down syndrome)

“Mild/moderate CID”: Characterized by an incomplete reduction in T-cell number or activity where part of the immune system’s ability to respond to infectious organisms is retained (e.g. partial DiGeorge syndrome, Ataxia Telangiectasia, Wiskott Aldrich syndrome, Early Purine Nucleoside Phosphorylase Deficiency)

^*^For each question, participants who did not answer were excluded when calculating the proportions

Knowledge and attitudes regarding immunization were generally similar between IDS and Immunologists (Additional file 1: Table S1). With regards to influenza vaccination, 100% of IDS that answered the question (17/17) considered inactivated influenza vaccine (IIV) to be very or somewhat safe in CID IEI patients, compared to 67% (10/15) of Immunologists who answered the question (p = 0.018). However, there were no differences in influenza vaccination recommendations (Table 4).

**Table 4:** Vaccine recommendations for B cell and CID IEI

Physician recommendation (N = 45)	Always/often/sometimes recommend % (n)	Rarely/never recommend % (n)	Did not answer % (n)
B cell			
*DTaP/Tdap*	84% (38)	2% (1)	13% (6)
*IIV*	82% (37)	2% (1)	16% (7)
*Influenza (LAIV)*	24% (11)	53% (24)	22% (10)
*MMR/ Varicella/ MMRV*	53% (24)	31% (14)	16% (7)
*Rotavirus*	31% (14)	40% (18)	29% (13)
CID			
*DTaP/Tdap*	73% (33)	2% (1)	24% (11)
*IIV*	67% (30)	9% (4)	24% (11)
*Influenza (LAIV)*	11% (5)	60% (27)	29% (13)
*MMR/ Varicella/ MMRV*	31% (14)	44% (20)	24% (11)
*Rotavirus*	18% (8)	56% (25)	27% (12)

*Imm* immunologists, *IDS* infectious disease specialists, *MMR* Measles/Mumps/Rubella Vaccine, *MMRV* Measles/Mumps/Rubella/Varicella Vaccine, *DTaP-Hib-IPV* Diptheria/Tetanus/acellular pertussis/Haemophilus Influenzae B/Inactivated Polio Vaccine, *DTaP* Diphtheria/Tetanus/Acellular Pertussis Vaccine, *TdaP* Tetanus/lower dose Diphtheria/Acellular Pertussis Vaccine, *IIV* Inactivated Influenza Vaccine, *LAIV* Live Attenuated Influenza Vaccine

A total of 41 physicians specified the number of IEI patients they saw in the prior 12 months. All (9/9) physicians who saw > 10 IEI patients considered live vaccines to be always/often/sometimes safe among mild/moderate CID patients, compared to 39% (9/23) of physicians who saw ≤ 10 IEI patients, with 9 physicians not answering this question (p = 0.002).

**Fig. 1:**
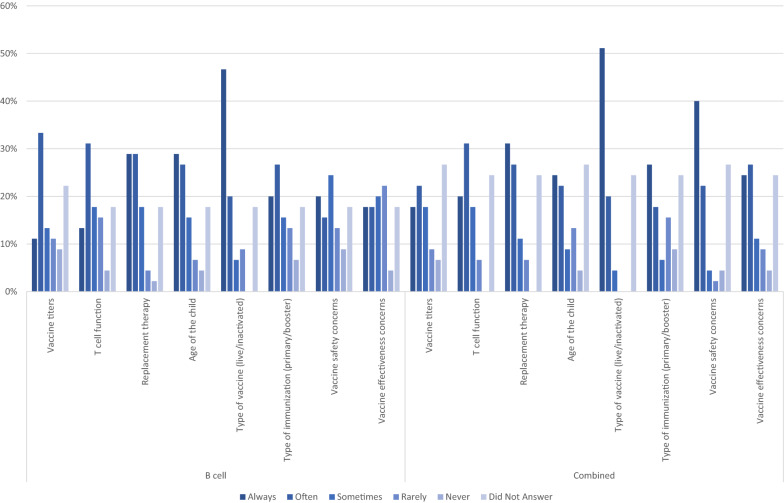
Factors influencing decisions for live/inactivated immunization for B cell and CID inborn errors of immunity reported by physician respondents (N = 45)

Factors that influenced physicians’ decision to recommend vaccination are shown in Fig. 1. The type of vaccine (live/inactivated) appeared to be the most important factor influencing immunization recommendation, implicated in the decision of 47% (21/45) and 51% (23/45) of physicians for B cell and CID patients, respectively. Vaccine safety was cited as a factor to “always” consider by 18/45 (40%) of physicians for CID patients and by 9/45 (20%) physicians for B cell patients. Immunologic functional testing also factored into physicians’ decision to recommend vaccination. Vaccine titres were listed as always/often a factor by 44% (20/45) of physicians recommending vaccination in B cell immunodeficient patients and in 40% (18/45) of physicians in recommending vaccination in CID. T cell function was listed as always/often a factor for 44% (20/45) of physicians in B cell immunodeficient patients and for 51% (23/45) physicians in CID (Fig. 1).

Correspondingly, most of the physicians who responded (62%%, 28/45 in B cell; 64%, 29/45 in CID) stated they recommended at least one type of immunologic investigation to guide vaccination recommendations. Of physician who reported recommending immunologic studies, the most common immunologic investigations recommended for B cell and CID immunodeficiencies, respectively were: lymphocyte subsets (53%, 15/28; 66% 19/29), quantitative immunoglobulins (53%, 15/28; 41%, 12/29), T cell proliferation assays (50%, 14/28; 66%, 19/29), and vaccine antigen-specific antibody titres (46%, 13/28; 38%, 11/29). The type of investigations ordered did not correlate with types of safety concerns expressed (e.g., those more concerned with safety were not more likely to recommend T cell subsets/proliferation assays) (Additional file 1: Table S2).

### Retrospective chart review

A total of 96 patients with IEI provided consent and were enrolled. Seventy-one percent of patients were male. The median age at IEI diagnosis was 15.5 months, (range: 0–134 months). Forty-five percent (43/96) of patients had predominantly antibody defects, 38% (36/96) had a CID with syndromic features, 14% (13/96) had CID without syndromic features and 4% (4/96) had defects in innate immunity or phagocytic function (Table 5 and Additional file 1: Table S3). Fifty-four percent (52/96) of these patients were classified as mild/moderate IEI.

**Table 5:** Demographics of participants in retrospective review (N = 96)

	Categories	N	%
Sex	Female	28	29
	Male	68	71
Age at diagnosis (months)	N	78*	
	Mean, SD	26.8, 28.6	
	Median, range	15.5, 0–134	
Type of primary immune deficiency	Immunodeficiencies affecting cellular and humoral immunity	13	14
	Combined immunodeficiency with associated or syndromic features	36	38
	Predominantly antibody deficiencies	43	45
	Defects in innate and phagocyte immunity	4	4
Age at enrollment (months)	N	96	
	Mean, SD	100.7, 50.1	
	Median, range	89.5, 22–259	
Age at enrollment	< 5 years	20	21
	≥ 5 years	76	79

^*^18 missing age at diagnosis

Overall vaccine completeness, and completeness of MMR/MMRV and DTaP-Hib-IPV at age two years and age seven years are shown in Table 6. Vaccine completeness was generally low and appeared to decrease from ages 2 to 7 for CID with and without syndromic features, and for predominantly antibody deficiencies. Only innate immunity defects had 100% (4/4 and 3/3) completeness at 2 and 7 years for DTaP/TdaP schedules. Age at diagnosis of IEI was not associated with vaccine completeness at age 7.

**Table 6:** Vaccine completeness at age 2 and 7 for IEI patients by age at diagnosis, type of primary immune deficiency, use of immunoglobulin replacement therapy (N = 95*)

		Overall vaccine completeness % (n)		Completeness of MMR/MMRV % (n)		Completeness of DTaP-Hib-IPV Vaccination % (n)	Completeness of DTaP/TdaP % (n)
		Age 2	Age 7	Age 2	Age 7	Age 2	Age 7
Age at IEI diagnosis (N = Age 2,Age 7)	Age at diagnosis at < 1 year (N = 28,13)	32% (9)	23% (3)	57% (16)	39% (5)	64% (18)	62% (8)
	Age at diagnosis 1 to < 4 years (N = 36,23)	36% (13)	4% (1)	75% (27)	30% (7)	53% (19)	26% (6)
	Age at diagnosis ≥ 4 years (N = 13,13)	46% (6)	15% (2)	85% (11)	62% (8)	69% (9)	46% (6)
	Age at diagnosis unavailable (N = 18,8)	61% (11)	13% (1)	89% (16)	25% (2)	89% (16)	63% (5)
Type of IEI	Immunodeficiencies affecting cellular and humoral immunity (N = 13,6)	23% (3)	17% (1)	62% (8)	17% (1)	54% (7)	50% (3)
	Combined immunodeficiency with associated or syndromic features (N = 35,21)	20% (7)	5% (1)	66% (23)	43% (9)	63% (22)	38% (8)
	Predominantly antibody deficiencies (N = 43,27)	58% (25)	15% (4)	81% (35)	41% (11)	67% (29)	41% (11)
	Defects in innate/phagocyte immunity (N = 4,3)	100% (4)	33% (1)	100% (4)	33% (1)	100% (4)	100% (3)
Immunoglobulin replacement therapy	Yes (N = 54,30)	39% (21)	10% (3)	70% (38)	33% (10)	67% (36)	33% (10)
	No (N = 40,27)	45% (18)	15% (4)	80% (32)	46% (12)**	65% (26)	58% (15)**

*MMR* Measles/Mumps/Rubella Vaccine, *MMRV* Measles/Mumps/Rubella/Varicella Vaccine, *DTaP-Hib-IPV* Diphtheria/Tetanus/acellular pertussis/Haemophilus Influenzae B/Inactivated Polio Vaccine, *DTaP* Diptheria/Tetanus/Acellular Pertussis Vaccine, *TdaP* Tetanus/lower dose Diphtheria/Acellular Pertussis Vaccine

^*^One patient was < 2y at time of assessment, so could not have age 2 completeness assessed

^**^N = 26

For inactivated vaccines, only patients diagnosed with IEI before 1 year of age had similar completeness at age 2 vs age 7 (age 2: 64% (18/28); age 7: 62% (8/13)). Those on immunoglobulin replacement were less likely to be complete for inactivated vaccines at age 7 (33%, 10/30), than those not on replacement (58% (15/26). Completeness of DTaP-containing vaccines at 2 years of age did not appear to differ by IEI type or receipt of immunoglobulin replacement.

For live attenuated vaccines, patients diagnosed with IEI before 4 years of age were less likely to be complete for MMR/MMRV at age 2, than those diagnosed after their 4th birthday. CIDs without syndromic features were less complete for MMR/MMRV vaccines at age 7 than predominantly antibody deficiencies or CID with syndromic features (CID: 17% (1/6); CID/syndromic: 43% (9/21); antibody deficiency 41% (11/27)).

### Influenza vaccination

Among patients < 5 years old at the time of enrollment, 12/20 (60%) had never received an influenza vaccine. Among patients ≥ 5 years old, 42/76 (55%) had missed receiving the influenza vaccine 3 times or more in the previous 5 years (Additional file 1: Table S4). Only 7% of participants were complete for influenza vaccine at age 7 (Additional file 1: Table S4).

### Immune workup prior to vaccination

Lymphocyte subsets were measured in 22/97 (23%) patients in the 6 months prior to live vaccination. The lowest CD4 + T cell count measured prior to receiving live vaccination for patients with combined and syndromic immunodeficiencies were 0.910 and 0.610 × 10^9^/L, respectively, while the lowest CD8 + T cell counts was 0.341 and 0.210 × 10^9^/L respectively (Additional file 1: Table S5). The most common serologic markers measured were diphtheria, tetanus and measles IgG, at 71% (68/96), 69% (66/96) and 65% (52/80 (16 not immunized for measles)) of patients, respectively (Additional file 1: Table S6). Lymphocyte proliferation studies were documented in 4 patients (4%; 4/96) in the six months preceding live vaccination.

## Discussion

To our knowledge this study is the first to characterize Immunologists’ and IDS’ practices regarding immunization in mild/moderate IEI patients, as well as to assess vaccine completeness in Canadian patients with IEI. We found that overall, the majority of physicians considered inactivated vaccines to be safe and somewhat effective in patients with mild/moderate B cell deficiencies and CIDs. Fewer than half indicated that live attenuated vaccines were safe or effective in both groups. Perceptions of safety, effectiveness and vaccine recommendations were generally similar among IDS and Immunologists, except for IIV in combined IEI, which some Immunologists viewed as unsafe. Physicians who saw a higher volume of IEI patients were more likely to recommend live attenuated vaccines. Among the factors physicians considered when making immunization recommendations, vaccine type and immunoglobulin replacement therapy were considered most often for patients with B cell IEI while vaccine type and vaccine safety were most frequently considered for patients with CID.

Upon retrospective chart review of 96 children from seven centres involved in the physician survey, most children with IEI were missing doses of both inactivated and live vaccines at ages 2 and 7 years, with completeness appearing to decrease between age 2 and age 7. Uptake of influenza vaccination was particularly low. While the physician survey focused on practices with regards to mild/moderate IEI and the retrospective review included patients with mild/moderate as well as more severe IEI, the majority of patients included in the review were classified as mild/moderate IEI where active immunization, especially influenza vaccination, would still be indicated. Though the patients reviewed were not all followed by the same physicians who responded to the survey, these findings of low vaccination uptake contrast with stated practices of physicians at the same centres. No consistent associations were noted between vaccination completeness and age at diagnosis, type or IEI or receipt of immunoglobulin replacement.

Physicians’ uncertainty regarding vaccine effectiveness in mild/moderate IEI could be related to variable data from vaccine immunogenicity studies in these patients. Vaccine immunogenicity studies have been conducted in patients with mild/moderate humoral IEI including common variable immune deficiency with class switched memory B cells, ≥ 0.4% of lymphocytes, transient hypogammaglobulinemia of infancy, IgA deficiency and IgG subclass deficiency. Results show that protective titres can be achieved post vaccination, albeit not consistently across all conditions or all antigens [31–40]. Both the Canadian Immunization Guide and US Centers for Disease Control and Prevention (CDC) guidelines state that in mild-moderate B cell IEI, vaccines can be effective though responses are likely attenuated [23, 24].

In mild/moderate combined IEI, Vakkilainen et al. found sustained antibody responses to MMR but decreased antibody responses to varicella vaccine when compared to healthy controls in patients with cartilage hair hypoplasia (CHH), though cell mediated responses varicella vaccine were sustained [6]. Physician concerns about safety of live attenuated vaccines may derive from their potential to cause vaccine-associated disease including vaccine derived rubella virus associated granulomas in CIDs, as well as from conflicting guidelines on their use in patients with mild-moderate IEI [7–24, 41]. For example, in partial CID, the CDC recommends avoidance of all live vaccines, while the Canadian Immunization Guide suggests that patients with a CD4 + T cell count > 0.500 × 10^9^/L and normal mitogen responses can receive MMR and univalent varicella vaccines [1, 23, 24, 26, 27].

Vaccine completeness in IEI patients in this study was much lower than overall Canadian averages during the same time period at both ages 2 and 7, with 76% of 2 year old Canadian children having received 4 doses of DTaP, and 90% having received at least one dose of measles vaccine in the 2013 Childhood National Immunization Coverage Survey (Total N = 4702) [42]. Studies in other immunocompromised hosts also show low vaccine uptake. Annunziata et al. reported 57.5% uptake of Hepatitis B vaccine and 52.2% uptake of pneumococcal vaccination in adult immunocompromised hosts in the USA [43]. Loubet et al. reported 49% uptake of pneumococcal vaccine in a large cohort of adult patients with secondary immune deficiency in France [44].

The reason for differences in perceptions of safety and effectiveness of influenza vaccination between subspecialists is unclear. Previous studies of influenza vaccine in IEI hosts have shown lower antibody responses, and in patients with B cell deficiencies, variable cell-mediated immune responses [34, 37, 40]. No serious adverse events following inactivated influenza vaccine were noted in these studies [32–36, 39]. Annual influenza vaccine is recommended by both national immunization guidelines and expert opinion even when patients are on immunoglobulin replacement to facilitate cell mediated protection against annual circulating strains of influenza virus [1, 23, 24, 33–37, 40]. Ballow et al. reported 76% and 74% influenza vaccine uptake for pediatric and adult patients with B cell defects (X-linked agammaglobulinemia, CVID, hypogammaglobulinemia) for the 2016–2017 influenza season in a survey of US IEI patients [45]. Cox et al. reported similar uptake of 76% for the 2019–2020 season in adult IEI patients receiving regular immunoglobulin replacement at a single Irish center [46]. Methodology for both studies was patient self-report rather than caregiver report validated by review of vaccine records as in our study.

Vaccine titres and T cell function were cited as factors influencing physician immunization recommendations for both B cell and CID patients. In practice, vaccine titres and lymphocyte proliferation prior to vaccination was documented in 28% (27/96). In the 6 months preceding live vaccination, only 23% (22/96) had documented lymphocytes subsets and 6% (6/96) had documented lymphocyte proliferation assays. Studies of partial DiGeorge syndrome (pDGS) and CHH offer some guidance regarding immunologic correlates of safety prior to considering live vaccination, but do not suggest when timing of immune workup should occur in relation to live vaccines [2–6]. Partial DGS patients with CD4 + T cells > 0.5 × 10^9^/L and adequate proliferative response to mitogens have safely received MMR vaccine [2–5]. In patients with CHH, varicella vaccine was given to patients with CD4 + T cells as low as 0.2 × 10^9^/L, with no serious adverse events reported and comparable cellular response to healthy controls [6]; however, humoral responses were decreased compared to controls. The same study did report sustained humoral response to MMR in these patients.

### Limitations

There are several limitations to this study. Not all pediatric centres in Canada that see patients with IEI participated in the retrospective review or survey and community allergist/immunologists were not included in the survey, and thus this study is not representative of all practitioners and IEI patients across Canada. However, all provinces with a pediatric tertiary care centre were represented in the survey. There was a high frequency of missing responses to some of the survey questions, perhaps due to the length of the survey, which may have introduced bias into the findings. Five-point Likert scale answers were collapsed into three categories for analysis so the heterogeneity of knowledge/perceptions in survey answers was simplified.

For the retrospective chart review, again the sample size is limited to sites who had active pediatric clinical Immunology service, as well as to sites that could approach patients for informed consent to be reviewed. As such, only patients from seven Canadian pediatric tertiary care centres were enrolled and the data is not representative of all patient with IEI in Canada. Additionally, data is not generalizable to more severe IEI phenotypes as those who died or went to hematopoietic stem cell transplant were excluded. Though multiple information sources were used to capture immunization records in the chart review (e.g., parental, public health, medical record), records may have been incomplete in some patients, particularly those who moved between provinces as each Canadian province maintains its own immunization records. The definition of vaccine completeness was not reflective of national standards, but rather was chosen to encompass the varying vaccination schedules in Canadian provinces. Data on influenza vaccination, even when cross-checked with public health immunization records, may be incomplete given the availability of influenza vaccine in a range of settings that may not all report vaccinations to a central database. Data on adverse events were not systematically collected and therefore we could not assess vaccine safety.

## Conclusions

There appears to be a gap between physicians’ stated vaccination practices for patients with mild to moderate IEI and vaccination uptake in that population. Physicians reported recommending inactivated vaccines to most patients and live vaccines to a subset of patients with IEI, yet most patients were under-vaccinated for all vaccines studied. This finding suggests a need to support catch-up vaccination for all eligible patients with IEI once safety of vaccination has been established for the patient based on immunologic evaluation. These are important clinical opportunities to prevent vaccine-preventable diseases in patients with IEI. Further research is needed to understand the factors contributing to under-vaccination of children with mild/moderate IEI and to identify effective targeted interventions to improve uptake.

## Supplementary Information

**Additional file 1: Table S1.** IDS (N=19) versus Immunologists (N=23) perceptions of vaccine effectiveness and safety. **Table S2.** Correlation of Physicians Concerns and Management Approach. **Table S3.** Specific Immune Deficiencies among Participants in the Retrospective Review. **Table S4.** Influenza Vaccine Completeness at Age 7. **Table S5.** Lymphocyte subset values prior live immunization, N=22. **Table S6.** Serology Studies Sent Post Vaccination.

## Data Availability

The datasets used and/or analysed during the current study are available from the corresponding author on reasonable request.

## Abbreviations

IEI: Inborn errors of immunity
Imm: Immunologists
IDS: Infectious disease specialists
MMR: Measles/Mumps/Rubella Vaccine
MMRV: Measles/Mumps/Rubella/Varicella Vaccine
DTaP-Hib-IPV: Diphtheria/Tetanus/acellular pertussis/Haemophilus Influenzae B/Inactivated Polio Vaccine
DTaP: Diphtheria/Tetanus/Acellular Pertussis Vaccine
TdaP: Tetanus/lower dose Diphtheria/Acellular Pertussis Vaccine
IIV: Inactivated Influenza Vaccine
LAIV: Live Attenuated Influenza Vaccine
CVID: Common variable immune deficiency
pDGS: Partial DiGeorge Syndrome
CHH: Cartilage hair hypoplasia
