# Supplementary material for: Physician vaccination practices in mild to moderate inborn errors of immunity and retrospective review of vaccine completeness in IEI: results from the Canadian Immunization Research Network
Source: Allergy Asthma Clin Immunol. 2022 Apr 9;18:32. doi: 10.1186/s13223-022-00667-1 (PMC8994318; doi:10.1186/s13223-022-00667-1)
Supplement: Supplementary file 1 — Additional file 1: Table S1. IDS (N=19) versus Immunologists (N=23) perceptions of vaccine effectiveness and safety. Table S2. Correlation of Physicians Concerns and Management Approach. Table S3. Specific Immune Deficiencies among Participants in the Retrospective Review. Table S4. Influenza Vaccine Completeness at Age 7. Table S5. Lymphocyte subset values prior live immunization, N=22. Table S6. Serology Studies Sent Post Vaccination. [file 13223_2022_667_MOESM1_ESM.docx]

**Additional Content**

SURVEY QUESTIONS

1. Which of the following best describes your practice?
2. Do you follow children (≤18 years) with primary B or T cell deficiency (PID) either as consultant or attending physician? (Theconsultant is defined as a physician that gives a one-time opinion on the patient’s management whereas the attending physician is in charge of regular follow up).
3. If yes, please specify on what qualification.
4. How many years have you been in practice?
5. Where is your main practice located? (Province/Territory)
6. Which of the following best describes your practice setting?
7. Is your practice setting university affiliated?
8. Does your practice setting have local practice guidelines for immunization of children with primary mild/moderate B cell deficiency?
9. Does your practice setting have local practice guidelines for immunization of children with primary mild/moderate T cell deficiency?
10. What information sources do you use to answer immunization-related questions?
11. In the past 12 months, how many patients with mild/moderate primary B cell deficiency did you see?
12. In the past 12 months, how many patients with mild/moderate primary T cell deficiency did you see?
13. In general, are vaccines administered to patients with mild/moderate B or T cell defects in your practice?
14. If no, how often do you refer your patients to get their immunizations?
15. If you refer your patients to get their immunizations, where do you refer them to?
16. It is safe to administer live vaccines to children with mild/moderate primary B cell defects. (Always, Often, Sometimes, Rarely, Never)
17. It is safe to administer inactivated vaccines to children with mild/moderate primary B cell defects. (Always, Often, Sometimes, Rarely, Never)
18. Do you recommend the following vaccines to children with mild/moderate primary B cell defects? (Always, Often, Sometimes, Rarely, Never)
    1. DTaP/Tdap
    2. Haemophilus influenza B (Hib)
    3. Live Influenza (LAIV)
    4. Inactivated Influenza (TIV)
    5. MMR (Measles-Mumps-Rubella)
    6. MMRV (MMR-Varicella)
    7. Conjugated Meningococcus C
    8. Quadrivalent conjugated meningococcal (ACYW135 )
    9. Unconjugated meningococcal vaccine
    10. PCV 13 (Conjugated Pneumococcus)
    11. 23 valent unconjugated Pneumococcal vaccine (PPSV 23)
    12. Rotavirus
19. Varicella
20. How frequently does each of the following influence your decision to recommend immunization for patients with mild/moderate primary B cell defects? (Always, Often, Sometimes, Rarely, Never)
    1. Current vaccine titers
    2. Other Immune function measured by labs (IgG, IgA and IgM levels, +/- IgG subclasses, +/- B cell count/memory B cell fraction)
    3. Current immunoglobulin replacement therapy
    4. Age of child
    5. Type of vaccine (live/inactivated)
    6. Type of immunization (primary/booster)
    7. Vaccine safety concerns
    8. Vaccine effectiveness concerns
21. How effective are the following vaccines in children with mild/moderate primary B cell defects? (Very effective, Somewhat effective, Don’t know, Somewhat ineffective, Very ineffective)
    1. DTaP/Tdap
    2. Haemophilus influenza B (Hib)
    3. Live Influenza (LAIV)
    4. Inactivated Influenza (TIV)
    5. MMR (Measles-Mumps-Rubella)
    6. MMRV (MMR-Varicella)
    7. Conjugated Meningococcus C
    8. Quadrivalent conjugated meningococcal (ACYW135 )
    9. Unconjugated meningococcal vaccine
    10. PCV 13 (Conjugated Pneumococcus)
    11. 23 valent unconjugated Pneumococcal vaccine (PPSV 23)
    12. Rotavirus
22. Varicella
23. How safe are the following vaccines in children with mild/moderate primary B cell defects? (Very safe, Somewhat safe, Don’t know, Somewhat unsafe, Very unsafe)
    1. DTaP/Tdap
    2. Haemophilus influenza B (Hib)
    3. Live Influenza (LAIV)
    4. Inactivated Influenza (TIV)
    5. MMR (Measles-Mumps-Rubella)
    6. MMRV (MMR-Varicella)
    7. Conjugated Meningococcus C
    8. Quadrivalent conjugated meningococcal (ACYW135 )
    9. Unconjugated meningococcal vaccine
    10. PCV 13 (Conjugated Pneumococcus)
    11. 23 valent unconjugated Pneumococcal vaccine (PPSV 23)
    12. Rotavirus
24. Varicella
25. Do you assess immunologic status before recommending immunization to children with mild/moderate primary B cell defects? (Always, Often, Sometimes, Rarely, Never)
26. If you answered always, often or sometimes, please specify what tests you order
27. Do you recommend annual live attenuated influenza vaccine (LAIV) in children with mild/moderate primary B cell defects? (Always, Often, Sometimes, Rarely, Never)
28. If sometimes, rarely or never, please choose your reason(s) not to recommend.
29. Do you recommend annual inactivated influenza vaccine in children with mild/moderate primary B cell defects? (Always, Often, Sometimes, Rarely, Never)
30. If sometimes, rarely or never, please choose your reason(s) not to recommend.
31. Do you check serology titers of vaccine antigens before recommending booster immunization to children with mild/moderate primary B cell defects? (booster immunization is defined as vaccines administered to children who have previously completed their primary vaccine series)
32. Do you ever check antibody titres after vaccination?

30. If yes, specify for which vaccine antigens and how often. (Always, Often, Sometimes, Rarely, Never)

- Measles
- Mumps
- Rubella
- Tetanus
- Diphtheria
- Varicella
- Other:

1. If you check antibody titres always or often, do you measure them more than once?
2. If yes, specify for which vaccine antigens
3. It is safe to administer live vaccines to children with mild/moderate primary T cell defects. (Always, Often, Sometimes, Rarely, Never)
4. It is safe to administer inactivated vaccines to children with mild/moderate primary T cell defects. (Always, Often, Sometimes, Rarely, Never)
5. Do you recommend the following vaccines to children with mild/moderate primary T cell defects? (Always, Often, Sometimes, Rarely, Never)
   1. DTaP/Tdap
   2. Haemophilus influenza B (Hib)
   3. Live Influenza (LAIV)
   4. Inactivated Influenza (TIV)
   5. MMR (Measles-Mumps-Rubella)
   6. MMRV (MMR-Varicella)
   7. Conjugated Meningococcus C
   8. Quadrivalent conjugated meningococcal (ACYW135 )
   9. Unconjugated meningococcal vaccine
   10. PCV 13 (Conjugated Pneumococcus)
   11. 23 valent unconjugated Pneumococcal vaccine (PPSV 23)
   12. Rotavirus
6. Varicella
7. How frequently does each of the following influence your decision to recommend immunization for patients with mild/moderate primary T cell defects? (Always, Often, Sometimes, Rarely, Never)
   1. Current vaccine titers
   2. Other Immune function measured by labs (IgG, IgA and IgM levels, +/- IgG subclasses, +/- B cell count/memory B cell fraction)
   3. Current immunoglobulin replacement therapy
   4. Age of child
   5. Type of vaccine (live/inactivated)
   6. Type of immunization (primary/booster)
   7. Vaccine safety concerns
   8. Vaccine effectiveness concerns
8. How effective are the following vaccines in children with mild/moderate primary T cell defects? (Very effective, Somewhat effective, Don’t know, Somewhat ineffective, Very ineffective)
   1. DTaP/Tdap
   2. Haemophilus influenza B (Hib)
   3. Live Influenza (LAIV)
   4. Inactivated Influenza (TIV)
   5. MMR (Measles-Mumps-Rubella)
   6. MMRV (MMR-Varicella)
   7. Conjugated Meningococcus C
   8. Quadrivalent conjugated meningococcal (ACYW135 )
   9. Unconjugated meningococcal vaccine
   10. PCV 13 (Conjugated Pneumococcus)
   11. 23 valent unconjugated Pneumococcal vaccine (PPSV 23)
   12. Rotavirus
9. Varicella
10. How safe are the following vaccines in children with mild/moderate primary T cell defects? (Very safe, Somewhat safe, Don’t know, Somewhat unsafe, Very unsafe)
    1. DTaP/Tdap
    2. Haemophilus influenza B (Hib)
    3. Live Influenza (LAIV)
    4. Inactivated Influenza (TIV)
    5. MMR (Measles-Mumps-Rubella)
    6. MMRV (MMR-Varicella)
    7. Conjugated Meningococcus C
    8. Quadrivalent conjugated meningococcal (ACYW135 )
    9. Unconjugated meningococcal vaccine
    10. PCV 13 (Conjugated Pneumococcus)
    11. 23 valent unconjugated Pneumococcal vaccine (PPSV 23)
    12. Rotavirus
11. Varicella
12. Do you assess immunologic status before recommending immunization to children with mild/moderate primary T cell defects? (Always, Often, Sometimes, Rarely, Never)
13. If you answered always, often or sometimes, please specify what tests you order
14. Do you recommend annual live attenuated influenza vaccine (LAIV) in children with mild/moderate primary T cell defects? (Always, Often, Sometimes, Rarely, Never)
15. If sometimes, rarely or never, please choose your reason(s) not to recommend.
16. Do you recommend annual inactivated influenza vaccine in children with mild/moderate primary T cell defects? (Always, Often, Sometimes, Rarely, Never)
17. If sometimes, rarely or never, please choose your reason(s) not to recommend.
18. Do you check serology titers of vaccine antigens before recommending booster immunization to children with mild/moderate primary T cell defects? (booster immunization is defined as vaccines administered to children who have previously completed their primary vaccine series)
19. Do you ever check antibody titres after vaccination?
20. If yes, specify for which vaccine antigens and how often. (Always, Often, Sometimes, Rarely, Never)

- Measles
- Mumps
- Rubella
- Tetanus
- Diphtheria
- Varicella
- Other:

1. If you check antibody titres always or often, do you measure them more than once?
2. If yes, specify for which vaccine antigens

**Table S1 IDS (N=19) versus Immunologists (N=23) perceptions of vaccine effectiveness and safety**

|  | **Vaccine Effectiveness** | | | |  | |  | **Vaccine Safety** | | | |  | |  |
| --- | --- | --- | --- | --- | --- | --- | --- | --- | --- | --- | --- | --- | --- | --- |
|  | Very/Somewhat Effective | | Very/Somewhat Ineffective | | Don’t Know | | Did not Answer | Very/Somewhat Safe | | Very/Somewhat Unsafe | | Don’t Know | | Did Not Answer |
| ***B CELL DISORDERS*** | Imm* | IDS* | Imm | IDS | Imm | IDS | Imm/  IDS | Imm | IDS | Imm | IDS | Imm | IDS | Imm/IDS |
| DTaP/Tdap | 60% (9) | 59% (10) | 13% (2) | 6% (1) | 27% (4) | 35% (6) | 8/2 | 93%  (13) | 100%  (17) | 0% (0) | 0% (0) | 7% (1) | 0%  (0) | 9/2 |
| Influenza (TIV) | 50% (7) | 53% (9) | 14% (2) | 6% (1) | 36% (5) | 41% (7) | 9/2 | 77%  (10) | 100% (17) | 15% (2) | 0% (0) | 8% (1) | 0% (0) | 10/2 |
| Influenza (LAIV) | 29% (4) | 35%  (6) | 21% (3) | 6% (1) | 50%  (7) | 59% (10) | 9/3 | 50%  (7) | 47% (8) | 29% (4) | 35% (6) | 21% (3) | 18% (3) | 9/2 |
| MMR/Varicella/MMRV | 40% (7) | 41% (7) | 20% (3) | 6% (2) | 40% (5) | 53% (8) | 8/2 | 64%  (9) | 47% (8) | 22% (3) | 47% (8) | 14% (2) | 6%  (1) | 9/2 |
| Rotavirus | 33%  (5) | 29%  (5) | 20%  (3) | 12%  (2) | 47%  (7) | 59% (10) | 8/2 | 50%  (7) | 53% (9) | 21% (3) | 24% (4) | 29% (4) | 24% (4) | 9/2 |
| ***CID*** | Imm | IDS | Imm | IDS | Imm | IDS |  | Imm | IDS | Imm | IDS | Imm | IDS |  |
| DTaP/Tdap | 60% (9) | 59% (10) | 20% (3) | 6% (1) | 20% (3) | 35% (6) | 8/2 | 87%  (13) | 100% (16) | 0% (0) | 0% (0) | 13% (2) | 0% (0) | 8/3 |
| Influenza (TIV) | 57%  (8) | 53% (9) | 14% (2) | 12% (2) | 29% (4) | 35%  (6) | 9/2 | 67%^1^  (10) | 100%^1^  (16) | 13% (2) | 0% (0) | 20% (3) | 0% (0) | 8/3 |
| Influenza (LAIV) | 21% (3) | 18% (3) | 21% (3) | 24% (4) | 47% (8) | 59% (10) | 9/2 | 7%  (1) | 31% (5) | 60% (9) | 50% (8) | 33% (5) | 19% (3) | 8/3 |
| MMR/Varicella/MMRV | 46% (6) | 29% (5) | 31% (4) | 23% (4) | 23% (3) | 47%  (8) | 10/2 | 20%  (3) | 19% (3) | 60% (9) | 75% (12) | 20% (3) | 6% (1) | 8/3 |
| Rotavirus | 15% (2) | 29% (5) | 23% (3) | 12% (2) | 62% (8) | 59% (10) | 10/2 | 7%  (1) | 31% (5) | 57% (8) | 50% (8) | 36% (5) | 19% (3) | 9/3 |

**For each question, participants who did not answer were excluded when calculating the proportions*

*For ID*

**^1^**p-value 0.02 – Chi squared

**Table S2 Correlation of Physicians Concerns and Management Approach**

| **Patients** | **Concerns** | **Tests reflecting the management approach**  *(=1 if at least one of the listed tests was requested by the physician)* | **Spearman correlation test^[[1]](#footnote-1)^** |
| --- | --- | --- | --- |
| B cell, Live vaccines  N=19 | Very/Somewhat effective  Very/Somewhat ineffective  Don’t know | Ig levels  vaccine titres pre / post immunization | Coefficient=0.42  p=0.06 |
| B cell, Inactivated vaccines  N=19 | Very/Somewhat effective  Very/Somewhat ineffective  Don’t know | Ig levels  vaccine titres pre / post immunization | Coefficient=0.32  p=0.17 |
| T cell, live vaccines  N=20 | Very/Somewhat safe  Very/Somewhat unsafe  Don’t know | lymphocyte subsets enumeration+/- lymphocyte function tests  T cell function/PHA | Coefficient=0.25  p=0.28 |

^1^Spearman correlation was undertaken to see if there was any correlation between investigations undertaken by physicians and attitudes towards vaccination (ex those more concerned with safety using T cell subsets/proliferation assays, those concerned with effectiveness using vaccine titres). No correlation was seen.

**Table S3 –** Specific Immune Deficiencies among Participants in the Retrospective Review

| **Immune Deficiencies in Retrospective Review (N=96)** | **N** |
| --- | --- |
| *Immunodeficiencies affecting cellular and humoral immunity* | |
| ADA Deficiency | 1 |
| Combined Immune Deficiency | 3 |
| DNA Ligase IV Deficiency | 2 |
| Hyper IgM Syndrome (CD40L Deficiency) | 1 |
| Severe Combined Immune Deficiency (No genetic mutation specified) | 4 |
| Hypogammaglobulinemia/T cell lymphopenia | 1 |
|  |  |
| *Combined immunodeficiency with or without associated or syndromic features* | |
| 22q11 deletion syndrome* | 26 |
| Ataxia Telangiectasia | 4 |
| Autosomal Dominant STAT3 Deficiency | 1 |
| Bloom Syndrome | 1 |
| Combined Immune Deficiency - BCL11B Mutation | 1 |
| Common Variable Immune Deficiency | 13 |
| Trisomy 21 | 1 |
| NEMO Deficiency | 1 |
|  |  |
| *Predominantly antibody deficiencies* | |
| APDS (Activated P13K-Delta Syndrome) | 1 |
| CARD 11 Gain of Function Mutation - BENTA | 1 |
| Hypogammaglobulinemia | 11 |
| Hypogammaglobulinemia/Specific Antibody Deficiency | 2 |
| IgG subclass Deficiency | 1 |
| IgM Deficiency | 1 |
| X-linked Agammaglobulinemia | 4 |
| Transient Hypogammaglobulinemia of Infancy | 8 |
| Transient Hypogammaglobulinemia of Infancy and MBL Deficiency | 1 |
| CTLA-4 Haploinsufficiency | 1 |
|  |  |
| *Defects of intrinsic and innate immunity and congenital defects of phagocyte number, function or both* | |
| Chronic Granulomatous Disease | 1 |
| MBL Deficiency | 1 |
| SCN2 Mutation | 1 |
| JAK1 Mutation | 2 |

**Table S4. Influenza Vaccine Completeness at Age 7**

|  | | **No** | | **Yes** | |
| --- | --- | --- | --- | --- | --- |
|  | **Categories** | **N** | **%** | **N** | **%** |
| Age at diagnosis (month) | Missing | 7 | 87.5 | 1 | 12.5 |
|  | < 1 year | 12 | 92 | 1 | 8 |
|  | 1 - <4 years | 22 | 96 | 1 | 4 |
|  | > 4 years | 12 | 92 | 1 | 8 |
| Type of Primary Immune Deficiency | Immunodeficiencies affecting cellular and humoral immunity | 4 | 67 | 2 | 33 |
|  | Combined immunodeficiency with associated or syndromic features | 21 | 100 | 0 | 0 |
|  | Predominantly antibody deficiencies | 25 | 93 | 2 | 15.4 |
|  | Defects in innate and phagocyte immunity | 3 | 75 | 1 | 25 |
| Immunoglobulin replacement therapy | No | 25 | 96 | 1 | 4 |
|  | Yes | 27 | 90 | 3 | 10 |
|  | Unknown | 1 | 100 | 0 | 0 |

**Table S5** Lymphocyte subset values prior live immunization, N=22

| **Diagnosis** | **CD3** (x10^9^/L) **(min-max, median)** | **CD4** (x10^9^/L) **(min-max, median)** | **CD8** (x10^9^/L) **(min-max, median)** | **CD19** (x10^9^/L) **(min-max, median)** |
| --- | --- | --- | --- | --- |
| Immunodeficiencies affecting cellular and humoral immunity | N=1  4.711 | N=2  0.910-3.163, 2.037 | N=2  0.341-1.211, 0.776 | N=2  0.151-1.481, 0.816 |
| Combined immunodeficiency with associated or syndromic features | N=11  1.300-4.347, 1.898 | N=11  0.610-2.913, 1.118 | N=11  0.210-1.430, 0.485 | N=11  0.552-2.764, 0.870 |
| Predominantly antibody deficiencies | N=8  1.160-6.732, 2.997 | N=8  0.850-4.896, 1.948 | N=8  0.160-1.428, 0.767 | N=8  0.130-12.444. 1.043 |
| Defects in Intrinsic or innate immunity | N=1  1.325 | N=1  0.732 | N=1  0.392 | N=1  0.785 |

Lymphocyte Subset Reference Ranges X 10^9/L, 5^th^ to 95^th^ percentile (Comans-Bitter J Pediatr 1997; 130:388-93)

- 9-15 months: CD3 1.6-6.7, CD4 1.0-4.6, CD8 0.4-2.1, CD19 0.6-2.7
- 2-5 years: CD3 0.9-4.5, CD4 0.5-2.4, CD8 0.3-1.6, CD19 0.2-2.1

**Table S6. Serology Studies Sent Post Vaccination**

| **Antigen** | **# of subjects immunized with vaccines containing the antigen** | **# of vaccinated subjects tested for antigen** | **# of unvaccinated subjects tested for antigen** |
| --- | --- | --- | --- |
| Measles | 80 | 52 (65%) | 4 |
| Mumps | 80 | 35 (44%) | 2 |
| Rubella | 80 | 37 (46%) | 6 |
| Varicella | 69 | 37 (53%) | 6 |
| Diptheria | 96 | 68 (71%) | 1 |
| Tetanus | 96 | 66 (69%) | 1 |
| Hib | 94 | 8 (9%) | 0 |
| Polio | 95 | 1 (1%) | 0 |
| Acellular Pertussis | 96 | 1 (1%) | 0 |
| Hepatitis B | 35 | 1 (3%) | 0 |
| Pneumococcus | 93 | 38 (41%) | 2 |

1. [↑](#footnote-ref-1)
